# Interaction and Inhibition of Dengue Envelope Glycoprotein with Mammalian Receptor DC-Sign, an In-Silico Approach

**DOI:** 10.1371/journal.pone.0059211

**Published:** 2013-03-18

**Authors:** Masaud Shah, Abdul Wadood, Ziaur Rahman, Tayyab Husnain

**Affiliations:** 1 Bioinformatics Research Laboratory, National Center of Excellence in Molecular Biology (CEMB), University of the Punjab, Lahore, Pakistan; 2 Computational Medicinal Chemistry Laboratory, Department of Biochemistry Abdul Wali Khan University, Mardan, Pakistan; Oak Ridge National Laboratory, United States of America

## Abstract

Membrane fusion is the central molecular event during the entry of enveloped viruses into cells. The critical agents of this process are viral surface proteins, primed to facilitate cell bilayer fusion. The important role of Dendritic-cell-specific ICAM3-grabbing non-integrin (DC-SIGN) in Dengue virus transmission makes it an attractive target to interfere with Dengue virus Propagation. Receptor mediated endocytosis allows the entry of virions due to the presence of endosomal membranes and low pH-induced fusion of the virus. DC-SIGN is the best characterized molecule among the candidate protein receptors and is able to mediate infection with the four serotypes of dengue virus (DENV). Unrestrained pair wise docking was used for the interaction of dengue envelope protein with DC-SIGN and monoclonal antibody 2G12. Pre-processed the PDB coordinates of dengue envelope glycoprotein and other candidate proteins were prepared and energy minimized through AMBER99 force field distributed in MOE software. Protein-protein interaction server, ZDOCK was used to find molecular interaction among the candidate proteins. Based on these interactions it was found that antibody successfully blocks the glycosylation site ASN 67 and other conserved residues present at DC-SIGN-Den-E complex interface. In order to know for certain, the exact location of the antibody in the envelope protein, co-crystallize of the envelope protein with these compounds is needed so that their exact docking locations can be identified with respect to our results.

## Introduction

**Figure 1:**
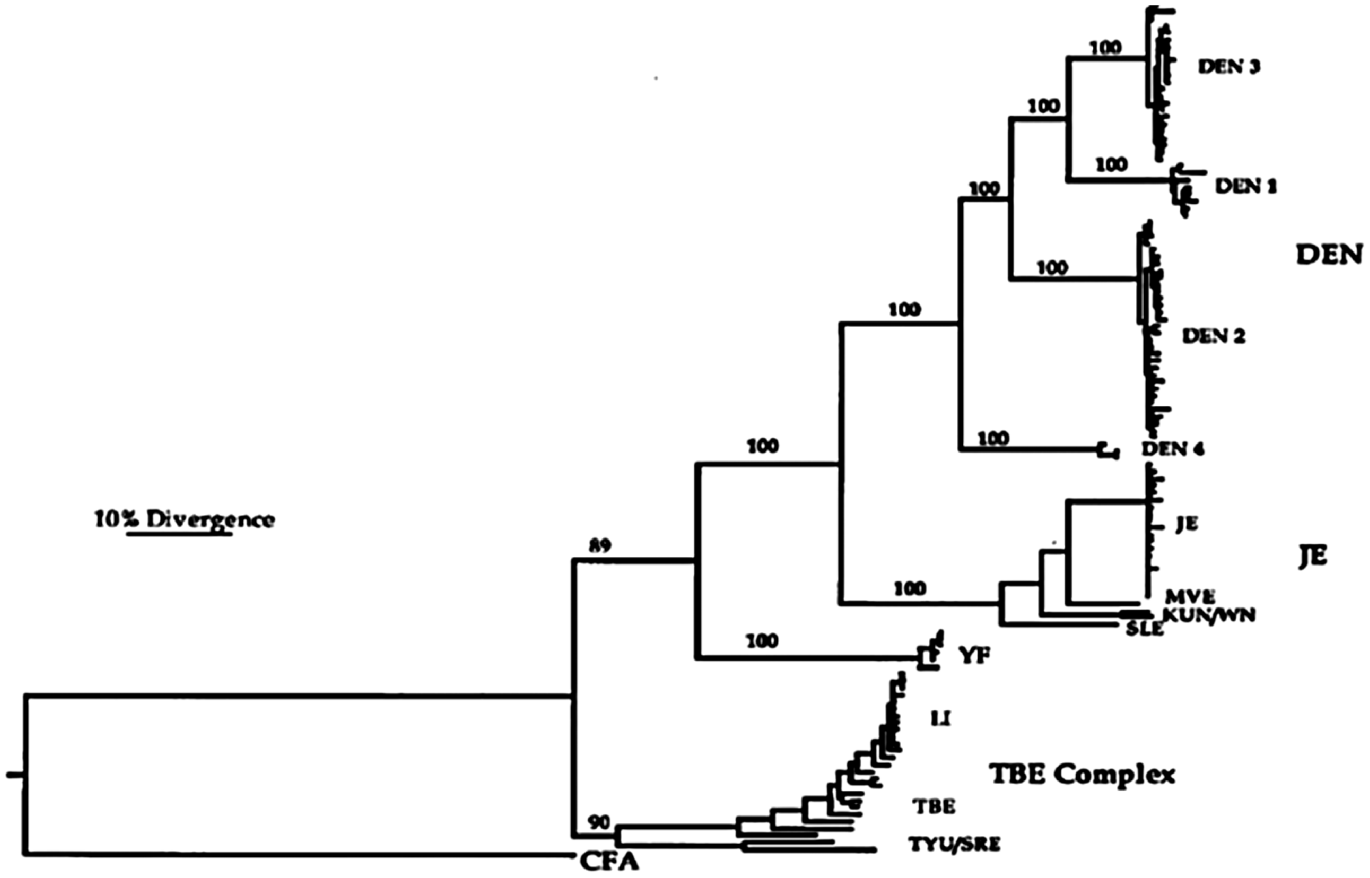
Maximum likelihood tree for the E gene from 123 flaviviruses . The tree is rooted by the sequence from *Aedes albopictus* cell fusion agent (CFA) virus.

Developing world is victim of largest vector-borne viral disease burden caused by the four serotypes of dengue virus (DENV) [1] and 50–100 million cases are reported yearly. Dengue virus has got its endemic in Pakistan and is circulating throughout the year [2]. Lifelong immunity against one serotype has been raised by DENV, while transient protection has been observed against other serotypes [3]. A greater risk for dengue hemorrhagic fever or dengue shock syndrome (DHF/DSS) is associated with different DENV serotype virus infection in the long term [4]. Viral uptake can be mediated by the presence of serotype cross-reactive and weakly neutralizing antibodies which enhance the infection of cells which bear F_cγ_ receptors; this phenomenon is termed as antibody-dependent enhancement (ADE) of infection. DENV belongs to flavivirus genus of the Flaviviridae family, including numerous other important human pathogens such as tick-borne encephalitis (TBE), yellow fever (YF), West Nile (WN) and Japanese encephalitis (JE) viruses [5]. The dengue virus was divided into four groups called serotypes based on antigenic properties. Subsequent evidence from molecular data reaffirmed this classification and also provided a clearer understanding of the phylogeny of the four serotypes: among the dengue viruses, DENV-4 diverged first from the common ancestor, followed by DENV-2, and finally DENV-1 and DENV-3 (figure 1) [6].

Lipid bilayer envelops the virus particles which are enclosed within an icosahedral scaffold of envelope glycoprotein E [7]. Receptor mediated endocytosis allows the entry of virions due to the presence of endosomal membranes and low pH-induced fusion of the virus [8]. Thus the major entry process of the flavivirus is through its envelope. Both the membrane fusion in the endosome and receptor binding are induced by two C-terminal transmembrane (TM) helices present in the Flavivirus E glycoprotein and are about 500 amino acids long. Crystallization of soluble fragment of Dengue virus E containing approximately 400 N-terminal amino acids has already been done [9]–[11].

**Figure 2:**
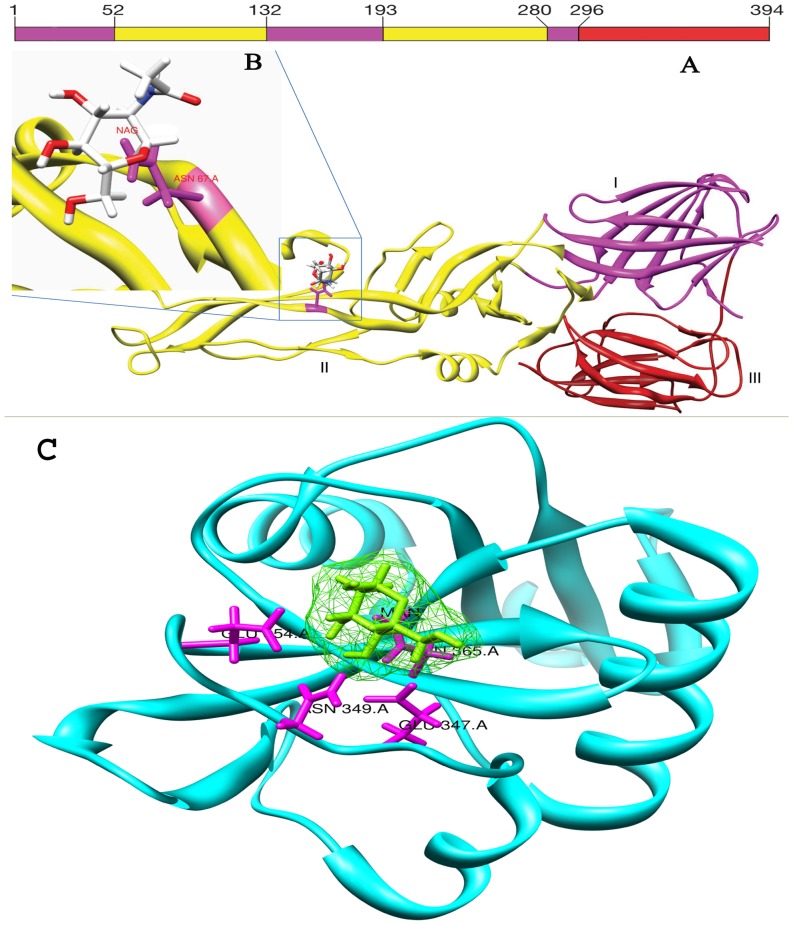
Structure of the monomer of dengue E soluble fragment (sE) in the mature virus particle. **A**, The three domains of dengue envelope, Domain I is magenta, domain II is yellow, domain III is red also indicated by bars above the figure. **B,** ASN 67-residue domain II links Dengue E to DC-SIGN Receptor on human dendritic cells. **C,** Structure of the carbohydrate recognition domain of human DC-SIGN attached by hydrogen bonds to mannose in green.

Three distinct domains (DI, DII, and DIII) constitute its structure (Figure 2a). DI containing N terminus is at the center, while DII and DIII are present at either side. Hydrophobic fusion loop has been displayed by DII and a conserved glycosylation site at residue ASN 67 (figure 2b).It has also been thought that DIII is involved in receptor binding [12].Despite intensive studies, relevant receptor(s)’ nature is not known at the surface of susceptible cells [13].

Reported DENV receptors include heat shock protein 70 (Hsp 70) and Hsp90 [14], GRP78 [15], laminin receptor [16], mannose receptor [17], CD14-associated protein [18], [19], DC-SIGN [20], [21] and various not entirely characterized polypeptides [22], [23] (Table 1).

**Table 1:** Dengue virus receptors reported I different studies.

Receptor	Properties	Cell/Tissue expression	Serotype
**Heparan sulfate**	Sulfated glycosaminoglycan	Vero cells, BHK-21 cells, SW-13 cells	DENV1–4
**nLc4Cer**	Glycosphingolipid	Vero cells, BHK-21 cells,	DENV1–4
**DC-SIGN/L-SIGN**	Dendritic cell-specific lectin,CD209	Dendritic cells, Macrophage	DENV-1-4
**Mannose receptor**	Protein with lectin activity	Macrophage	DENV-1-4
**HSP70/HSP90**	Expression on plasma membrane	HepG2 cells, SK-SY-5Y cells, Macrophage	DENV-2
**GRP78**	Expression on plasma membrane Chaperon	HepG2 cells	DENV-2
**Laminin receptor**	High-affinity laminin receptor	PS Clone D cells, HepG2 cells	DENV-1-3
**CD14-associated protein**	Protein associated with LPS receptor	Monocyte, Macrophage	DENV-2

DC-SIGN is the best characterized molecule among the candidate protein receptors and is able to mediate infection with the four serotypes of DENV. DENV replication occurs in the skin followed by intradermal injection of DENV-2 in mice through an infected mosquito bite [24]. It has been believed that the primary DENV target cells in the skin are Langerhans cells or immature dendritic cells (DC) [25]–[28]. Pathogen capturing is quite efficient in case of immature DC while mature DC is quite resistant to infection [29]–[31]. DC-SIGN is a tetramer belonging to calcium-dependent C-type lectin family. It is composed of four domains: a cytoplasmic domain containing a dileucine motif which is responsible for internalization and signaling, a trans-membrane domain, seven to eight extracellular neck repeats implicated in the oligomerization of DC-SIGN and a carbohydrate recognition domain (CRD) (Figure 2c) [32].

The CRD recognizes fucose and high-mannose N-glycans containing blood group antigens [33], [34]. Using DC-SIGN-specific monoclonal antibodies, its expression has been found in immature myeloid DCs in skin, intestine, lung, liver, placenta and lymph nodes [35]–[37]. The participation of DC-SIGN in dengue virus infections was shown by competition assays employing either monoclonal antibodies against DC-SIGN or soluble DC-SIGN to inhibit DENV infection [38], [39]. Important cellular functions show the involvement of DC-SIGN [40].Cellular functions of DC-SIGN will also be prevented by blocking HIV transmission using antibodies against DCSIGN [41]–[43]. Therefore, a powerful alternative may be provided by peptide or ligand based inhibiters against dengue envelope protein to block the interaction of DC-SIGN–Dengue.

The identification of this and other receptors can open a way to the development of specific, receptor-based prophylaxis and therapy as well as, potentially, the early genetic identification of individuals at increased risk of developing dengue fever or, more importantly, dengue hemorrhagic fever or dengue shock syndrome. This study focus on identifying conserved residue (may or may not be epitopes) of Dengue virus serotypes 1–4 that interact with DC-SIGN receptors and designing peptide and ligand based inhibiters against those interfacing residues to block dengue and DC interaction.

## Materials and Methods

### Sequence Alignment and Epitopes Prediction

ClustalW based on progressive alignment methods available in MEGA 5 [44] and BioEdit [45] were used for multiple sequence alignment to find conservancy in domain I, II, III, of Envelop Glycoprotein of dengue virus serotype 1–4. 400 (100 for each serotype) non-redundant amino acid sequences were retrieved from UniProt database for alignment studies (http://www.uniprot.org/help/uniprotkb). All images were resized and developed by Adobe Photoshop CS3.

### B Cell Epitope Prediction

The ABCpred server based on partial recurrent neural network with a single hidden layer was used to predict B cell epitopes [46]. The physiochemical properties including turns, exposed surface, polarity, accessibility, flexibility/mobility and hydrophilicity were used and combination of the B-cell epitopes properties were predicted using Becpred Server [46]. Propensity scales were used for the prediction of calculations for each of the 20 amino acids. (http://www.imtech.res.in/raghava/bcepred/index.html).

### Assessment of the Models

High resolution X-Ray 3D crystal structure of dengue virus serotype 2 (pdb code: 1OK8), DC-SIGN CRD (pdb code: 1SL4) and Antibody 2G12 (pdb code: 3OAU) were retrieved form pdb database. We employed ProQ [47], ModFOLD [48] in order to check the quality of protein geometry. ProQ ranks models on the basis of LGscore (>1.5–>4) and MaxSub (>0.1–0.8) where the lower values corresponds to fairly good model and high values for extremely good models. LGscore is above 5 and MaxSub score is above 0.35 for all the models in this study. Likewise ModFOLD ranks models by P-values and global model quality scores range between 0 and 1. Scores less than 0.2 indicate there may be incorrectly modeled domains and scores greater than 0.4 generally indicate more complete and confident models. Our selected models have higher global model quality scores than 0.4 and are considered good candidates for docking purpose.

### Protein-protein Docking

We used unrestrained pairwise rigid body docking for dc-CRD-DEN-E and 2G12-envelope protein of dengue virus. Pre-processed the PDB coordinates of dengue envelope glycoprotein (PDB ID: 1OK8); DC-SIGN CRD domain (PDB ID: 1SL4) and Antibody 2G12 (PDB ID: 3OAU) were used prior than docking procedure for energy minimization through AMBER99 force field distributed in MOE 2011.10 software with 0.05 gradient on default parameters.

Protein-protein docking software, ZDOCK [49], was used to perform molecular docking, to predict and assess the interactions in dengue envelope and 2G12 antibody. This program ranks the 100 most probable predictions on the basis of electrostatic complementarity, hydrophobicity and geometry of the molecular surface out of thousands of candidates. Further qualifications including (i) predicted conserved epitopic residue in interaction sites; (ii) residue conservation of the interaction sites; (iii) Participation of the DC-MAN and antibody 2G12-MAN binding residue in selected complexes were implemented in the finally selected docked complexes from the top 100 predictions. A unique solution was obtained through this three-step filtering method. Energy minimization was applied on the final solutions obtained. Hydrogen was added to each atom (residues of Asp, Glu, Lys and Arg were considered ionized, whereas His residues were considered to be neutral) using AMBER99 force field distribution available in the MOE package in order to assign partial charges. PISA (protein interfaces, surfaces and assemblies service) at the European Bioinformatics Institute [50] was used to calculate the buried surface interaction area of all the docked models. UCSF Chimera package [51] and the nonlinear Poisson-Boltzmann equation with the APBS tools plugin for Pymol were used to calculate molecular electrostatics, RMSD and structural superimpositions.

## Results

### Epitope Prediction and its Conservancy Identification in Dengue Virus E Protein

An important role is played by B-cell epitope in synthetic vaccine design and also in humoral response. Sequence-based methods are limited to the prediction of continuous epitopes. The majority of the sequence-based methods assume that epitopes have to be accessible for antibody binding and, hence, are based on using epitope properties related to surface exposure. The epitopes predicted by ABCpred database in dengue virus envelope protein are listed in Table 2 and shown in supplementary data (S Figure 1 in supplementary file 1). Conserved antigenic epitopes present in the dengue E protein, were found by epitope conservancy identification. Average antigenicity properties were determined for B cell epitopes of Dengue Envelope protein.

**Table 2:** Predicted B-cell epitope and its conservancy.

Rnk	Predicted EpitopesIn Den 1–4	Start position	Score	Epitope % conservancy			
				Den 1	Den 2	Den 3	Den 4
**1**	DSPVNIEAEPPFGDSYII	362	0.91	72.22	100	72.22	72.22
**2**	RGWGNGCGLFGKGGIVTC	99	0.88	83.33	100	88.89	94.44
**2**	SYSMCTGKFKVVKEIAET	298	0.88	72.22	100	55.56	66.67
**3**	VITPHSGEEHAVGNDTGK	140	0.87	50.00	94.44	50.00	61.11
**4**	LTNTTTESRCPTQGEPTL	65	0.86	78	94	72.22	66.67
**4**	AKNKPTLDFELIKTEAKQ	35	0.86	72.22	100	83.33	77.78
**4**	TFKNPHAKKQDVVVLGSQ	239	0.86	83.33	100	88.89	83.33
**5**	RKYCIEAKLTNTTTESRC	57	0.85	77.78	100	72.22	61.11

The predicted B cell epitopes are ranked according to their score obtained by trained recurrent neural network. Higher score of the peptide means the higher probability to be as epitope. All the peptides shown here are above the threshold value chosen.

Using multiple physiochemical parameters and different analysis software, Epitopes at 65–83 and 57–74 amino acids are consider good for Dengue E protein. Physiochemical properties of B cell epitope including turns, exposed surface, polarity, accessibility, flexibility, and hydrophilicity are shown in supplementary data (S Figure 2 in supplementary file 1). The graph uses a scale normalized between +3 to −3. High values give rise to peaks, whereas valleys correspond to negative properties of the protein. The peak of the amino acid residue segment above the threshold value (2 to 2.5) is considered as predicted B cell epitope. The amino acid sequences falling in conserved region covering ASN 67 and having higher binding scores have a higher possibility of showing antibody response and thus are considered as candidate epitopes in Den-E-2G12 docking studies.

### Docking

Protein-protein docking procedure is extremely computationally oriented method. Quality of the docking methods tells the reliability of docking results. For the verification of the prediction confidence of Den-Envelope-DC-SIGN and Den-Envelope-2G12 interaction of ZDOCK, we unrestrainedly inputted domain III of dengue virus serotype 1 and Mab 4E11, with known heterodimeric crystal structures, as test cases [52].

The experimentally proved interaction of dengue virus serotype 1 and Mab 4E11 complexes were found in the top 100 solutions of ZDOCK ranked on the bases of Z scoring. Table 3 showing interfacing residues in both test cases. This test points out the reliability and feasibility of Z-DOCK used in Dengue E protein docking with DC-SIGN and 2G12; and we used them in further docking calculations. To explain how Dengue envelope protein binds with the 2G12 antibodies and avoids their attachment with DC-SIGN, and also how dengue envelope protein bind to DC-SIGN, which mediates cell attachment and entry, we conducted unrestrained rigid-body docking of DC-SIGN-DEN-E and DEN-E-2G12.

**Table 3:** ZDOCK confirmation results.

**No.**	**Complexes**		**Interfacing residues**
1	DomainIII-mab (PDB)	Domain III	**S305F306K307L308** E309 **K310 E311 V312** K325 E327 D360 K361 **E362** K363 P364 K385 L387 **K388** L389 **S390** W391
		mab	L4 G27 F28 N29 **K31** T33 **Y34** R51 **D53** A55 **R99** G100 W101 **E102** A105 **Y106R163Y164** G165 **N166Y185R186** N189 **E191** S192
2	DomainIII-mab (test)	Domain III	S305 **F306 K307 L308 E309 K310 E311 V312** K325 E327 T329 K361 **E362** K363 P364 K385 L387 **K388** L389 **S390** W391
		mab	L4 G27 F28 N29 **K31** T33 Y34 R51 D53 A55 **R99** G100 W101 E102 A105 **Y106 R163 Y164** G165 **N166** Y185 **R186** S188 **N189E191** S192

Table enlists interfacing residues in both test (ZDOCK Docked) and PDB crystal structure of (pdb cod 3UZQ). Bold residues are involved in hydrogen bonding.

**Figure 3:**
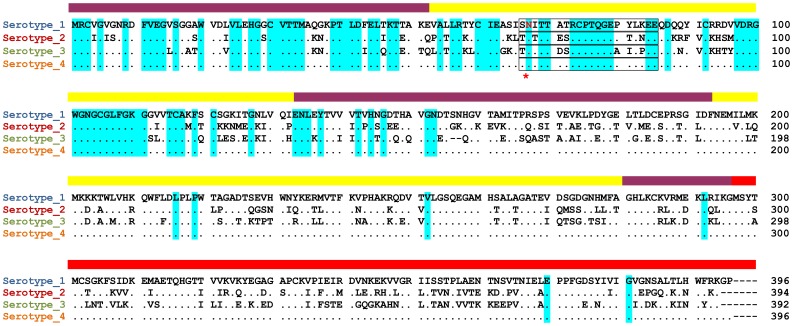
Structure-based alignment of the 400 non-redundant amino acid sequences of E proteins from Dengue Virus serotype 1–4. Dots indicate amino acid identities; dashes show gaps. The domains are indicated by a colored bar as in Fig. 1. The conserved glycosylation site in domain II is indicated by a red asterisk and red lettering. Residues in box indicates epitopic region covering ASN 67. Residues that are conserved in All four serotypes of Dengue virus are colored in sky blue.

100 most feasible models were obtained from docking of unbound monomer components. We selected 100 candidates from the best docking solution for each complex on the basis of following criteria: (i) models having no intersection in domain II of dengue E protein were omitted; (ii) only those shared models were included where the binding region is explained with experimental data [53], [54]; (iii) in Den-E-2G12 complexes only those models were selected which shows interaction in conserved epitopic residues of Dengue envelope covering ASN67 (Figure 3). Unique solution was obtained through this three-step filtering method.

The ZDOCK ranking of all the optimal models along with buried surface interaction area are shown in supplementary data (S Tables 1, 2 in supplementary file 2). We have selected the complex having highest rank from Table 2 and subjected this model to the identification of residual interface and energy minimization.

### Pair Wise Docking of Den-E-DC-SIGN and Den-E-2G12 Complex

The structural interfaces between Dengue envelope and DC-SIGN CRD domain have been described previously by electron microscopy [55]. However, there molecular level interaction is not yet available. The unavailability of these Den-E-DC-SIGN complexes is a hindrance to understand that how DC-SIGN regulate virus attachment and their entry into cells. In relation to the before solved crystal structure of DC-SIGN (1SL4) [54] its possible interactions with Den-E (1OK8) [57] have been identified by protein-protein docking method. Using the same procedure we also find interaction between 2G12 (3OAU) [58] and Den-E (1OK8) to design a therapeutic strategy for dengue fever by blocking dengue virus envelope conserved epitopic residues interacting with human receptors DC-SIGN.

### Docking Results of DC-SIGN-Envelope Glycoprotein

ZDOCK ranks model on the basis of best average so the highest ranked model was accepted as the optimal model. We evaluated interface surface areas and the interacting residues from all of the resulting docking models. Experimentally validated residues and amino acids, charged residues and interchain hydrogen bonds from the interfacial region were also evaluated, which are shown in Table 4.

**Table 4:** List of interfacing residues between Den-E-DC-SIGN-CRD complexes.

#	Complexes		interfacing residues	ISA*, Å^2^	HPI**
1	Den-E-DC-SIGN-CRD	Den-E	L65 **T66 N67 T69 T68** T70 ***E71*** **R73 C74 Q77** T81 L82 N83 E84 Q86D87*R89* F90 G102 **N103 G104**M118 T120 C121 **K122** N124 **T226** **Q227**G228 S229 N230 ***H244*** ** A245** ***K246K247***D249	934	0.44±0.56
		DC-SIGN-CRD	F262 Q264 G265 N266**N272Q274**Q290 K295 S296A297 E298 N301 **N311**R312 **F313** D331 **G332** S333 P334 L336 P337 S338 F339 **Q341**N344*R345* G346***E347*** **N349** V351 ***Q354*** ** S360** N365***A366*** D367 **K368** L371F374 K378 S380 A381 **S383** C384 ***E299*** ** Q328 Y342**	911	0.65±0.74

Residues involved in the formation of H-bond are in bold while both bold and italic are involved in salt bridges.

* Total change in surface area of the interface for a given chain. DSSP program [59].

** Hydrophobicity index (mean). AAIndex database [60].

**Figure 4:**
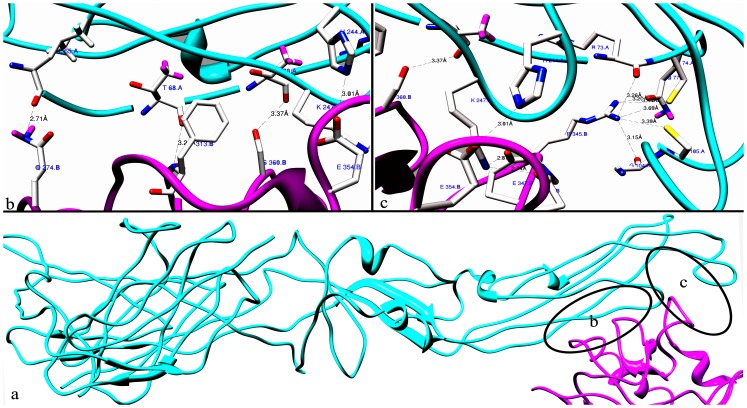
Dengue envelope glycoprotein and DC-SIGN interface. (**a**) Den-E-DC-SIGN Complex represented as a ribbon diagram are shown inCyan and Pink, respectively. (**b**) The Dengue envelope (chain A)-DC-SIGN binding interface. Side chains of the amino acids contributing to hydrogen bonding formation (indicated byblack dotted lines) are represented by a stick model with the residue names and numbers shown. (**c**) The Den-E (chain A)-DC-SIGN binding interface is also represented in a similar fashion as (**b**).

The buried surface at the border of the final Den-E-DC-SIGN complex is 908.0 A^o2^ covering 39% of the surface area of the complex. At the interface of Den-E-DC-SIGN complex, sixteen Hydrogen bonds are present (S Table 3 in supplementary file 2). The predominant interactions in Den-E-DC-SIGN complex are formed by hydrogen bonds of DC-SIGN ASN 272 with MAN, glycosidically attached to Den-E ASN-67 and six salt bridges present in different residues of both participating protein molecules (Figure 4). Lysine 247 of Den-E forms three hydrogen bonds with GLU 347, ASN 349 and ASP 366 one each of DC-SIGN. ARG 345 of DC-SIGN-CRD is attached by five different hydrogen bonds to THR 70, ARG 73, CYS 74, GLN 77, GLY 104 and CYS 105 of Den-E. Seven salt bridges are found in Den-E-DC-SIGN complex. Two salt bridges are present in LYS 247 and ASP 366, one between LYS 247 and GLU 354, one between LYS 247 and GLU 347 and one between HIS 244 and GLU 354 of Den-E and DC-SIGN CRD respectively. On the basis of our current model, it has been proposed that blocking ASN 67 of Den-E and its surrounding conserved interfacing residues in Den-E-DC-SIGN complex can block dengue virus attachment to its candidate receptor.

### Docking Results of Den-E-2G12 Complex

**Figure 5:**
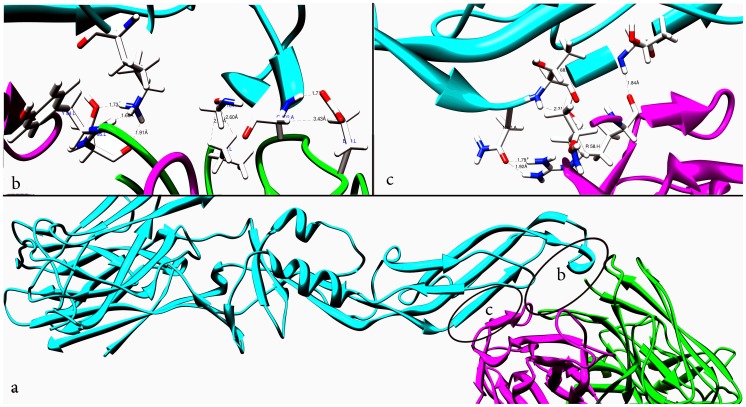
Dengue envelope glycoprotein and 2G12 antibody interface. (**a**) Den-E-2G12 Complex represented as a ribbon diagram are shown in Cyan and Pink and Green, respectively. (**b**) The Dengue envelope (chain A) and 2G12 light chain binding interface. Side chains of the amino acids contributing to hydrogen bonding formation (indicated by black dotted lines) are represented by a stick model with the residue names and numbers shown. (**c**) The Den-E and 2G12 heavy chain binding interface is also represented in a similar fashion as (**b**). (**d**)NAG sugar present at Den-E-2G12 interface.

444.3 A^o2^ with ten hydrogen bonds and five salt bridges is the buried surface area at the interface of the Den-E-2G12 H chain. Light chain of 2G12 has seven hydrogen bonds at the interface with 419 A^o2^buried surface area. Residues of Den-E involved in interaction with 2G12 are present in conserved epitopic region as predicted and confirmed in this study Figure 3. Residues at the interface of Den-E-2G12 complex are listed in table 5. SER 95 and TYR 94 of 2G12 light chain are linked by hydrogen bond with LYS 246 of Den-E (Figure 5). GLU-L 30 and VAL-L 2 are linked by hydrogen to GLY 102 and 104 of Den-E. Heavy chain of 2G12 is kinked by ten hydrogen bonds to Den-E. ASP 106 of heavy chain has two hydrogen bonds with LYS 247 of Den-E (S Table 4 in supplementary file 2).

**Table 5:** List of interfacing residues between Den-E-2G12 complexes.

#	Complex		interfacing residues	ISA*, Å^2^	HPI**
1	Den-E-2G12	Den-E	***K64*** L65 **T66N67 T68T70** ***G71***R89 M 118 F 119 T120 *K * ***122*** V 250 V 252 *K 58* **G102**E 126 K 128 K 202 D 203 *H244*D 225 **T 226** Q 227 G 228 S 229**K246** ***K247*** S 274	445	0.47±0.50
		2G12 H	S30 A31 T33 W47 S50 S52 T53 S54 **T56** Y57 **R58**D59**L65** L104 S 105 **D106** N 107	492	0.52±0.63
		2G12 L	**V 2** Q27 S28 **E30** T31W 32 **K50** T69 A92 **G93 Y 94 S95**	504	0.59±0.83

Residues involved in the formation of H-bond are in bold while both bold and italic are involved in salt bridges.

* Total change in surface area of the interface for a given chain. DSSP program [59].

** Hydrophobicity index (mean). AAIndex database [60].

Heavy chain ARG 58 have three hydrogen bond with dengue virus conserved glycosylation site ASN 67 and THR 68, which is also present in predicted epitopes for dengue virus serotypes 1–4. LYS-L 65 is attached by tow hydrogen bonds to THR 70 and GLU 71 of dengue envelope predicted epitopic region. Heavy chain ASP 106 is attached by three salt bridges to LYS 247 of Den-E. LYS-H 65 is linked by salt bridge to GLU 71 of dengue envelope. It has been proposed that generally neutralizing antibody 2G12 recognizes a unique group of high-mannose oligosaccharides and other conserved residues on Dengue virus envelope protein. wet lab confirmation of this and other such antibodies may be used to develop a single shot therapy for all four serotypes of dengue virus, because it recognize conserved epitopes as well as glycosylation site involved in dengue virus attachment to DC-SIGN.

Total RMSD for all final docked complexes and non-docked single PDB crystal structures was calculated by Needleman-Wunsch method based on BLOSUM62 Substitution Matrix [61] in chimera. Total RMSD between 132 atom pairs of DC-SIGN when superimposed over its homolog in Den-E-DC-SIGN complex is 0.910 Å. Total RMSD for each of three chains (A, H, L) in 2G12-Den-E complex was also calculated by chimera. Heavy chain of 2G12 in complex with Den-E deviate from its non-treated pdb crystal by 0.945 Å after docking. Light chain in dock complex deviate by 1.239 Å from light chain of pdb non minimized crystal of 2G12(3OAU).

## Discussion

During the entrance of enveloped viruses into the cells, central molecular event is played by membrane fusion. Viral surface proteins are the key agents for this process which help in facilitating bilayer fusion and conditions of target cell interactions with viruses are triggered by it.

DC-SIGN is an attractive target in Dengue virus transmission because of its important role of interference in Dengue virus Propagation. HIV-1 captured by DCs has been inhibited by blocking antibodies against DC-SIGN but it has also shown interference with the immunological role of DC-SIGN [41], [42].

One CRD monomer was found to bind to two glycosylation sites at Asn67 of two neighboring glycoproteins in each icosahedral asymmetric unit, leaving the third Asn67 residue vacant [55]. Our finding shows predominant interactions in Den-E-DC-SIGN complex are formed by hydrogen bonds of DC-SIGN ASN 272 with MAN, glycosidically attached to Den-E ASN-67 and six salt bridges present in different residues of both participating protein molecules. Lysine 247 of Den-E forms three hydrogen bonds with GLU 347, ASN 349 and ASP 366 one each of DC-SIGN-CRD. ARG 345 of DC-SIGN-CRD is attached by five different hydrogen bonds to THR 70, ARG 73, CYS 74, GLN 77, GLY 104 and CYS 105 of Den-E.

Inhibitors targeting dengue virus envelope protein to avoid DC-SIGN arrest seem acceptable to restrict with dengue dissemination. Additional knowledge about DC-SIGN–Den-E interaction is required for the formation of these inhibitors. Detailed interaction of DC-SIGN has been investigated here with Dengue virus envelope glycoprotein using a computer based protein-protein docking approach. Envelope residues at the interface of DC-SIGN-Den-E complex are conserved in all four serotypes of dengue virus and are found in conserved continuous predicted B cell epitopes.

Based on these interactions it was found that antibody successfully blocks the glycosylation site ASN 67 and other conserved residues present at DC-SIGN-Den-E complex interface. Heavy chain ARG 58 have three hydrogen bond with dengue virus conserved glycosylation site ASN 67 and THR 68, which is also present in predicted epitopes for dengue virus serotypes 1–4. LYS-L 65 is attached by tow hydrogen bonds to THR 70 and GLU 71 of dengue envelope predicted epitopic region. Heavy chain ASP 106 is attached by three salt bridges to LYS 247 of Den-E. LYS-H 65 is linked by salt bridge to GLU 71 of dengue envelope. It has been proposed that generally neutralizing antibody 2G12 recognizes a unique group of high-mannose oligosaccharides and other conserved residues on Dengue virus envelope protein. In order to know for certain, the exact location of the antibody in the envelope protein, co-crystallization of the envelope protein with these compounds is needed so that their exact docking locations can be identified with respect to our results.

### Conclusion

Critical agents of cell entry process are viral surface proteins, primed to facilitate cell bilayer fusion. The important role of Dendritic-cell-specific ICAM3-grabbing non-integrin (DC-SIGN) in Dengue virus transmission makes it an attractive target to interfere with Dengue virus Propagation.

Detailed interaction of DC-SIGN has been investigated here with Dengue virus envelope glycoprotein. Envelope residues at the interface of DC-SIGN-Den-E complex are conserved in all four serotypes of dengue virus and are found in conserved continuous predicted B cell epitopes. Based on these interactions it was found that antibody in this study successfully blocks the glycosylation site ASN 67 and other conserved residues present at DC-SIGN-Den-E complex interface. In order to know for certain, the exact location of the antibody in the envelope protein, co-crystallization of the envelope protein with these compounds is needed so that their exact docking locations can be identified with respect to our results.

## Supporting Information

File S1 Includes Figures S1 and S2.
(DOCX)

File S2 Includes Tables S1 and S2.
(DOCX)
