# Supplementary material for: Interaction and Inhibition of Dengue Envelope Glycoprotein with Mammalian Receptor DC-Sign, an In-Silico Approach
Source: PLoS One. 2013 Mar 18;8(3):e59211. doi: 10.1371/journal.pone.0059211 (PMC3601059; doi:10.1371/journal.pone.0059211)
Supplement: File S1 — Includes Figures S1 and S2. (DOCX) [file pone.0059211.s001.docx]

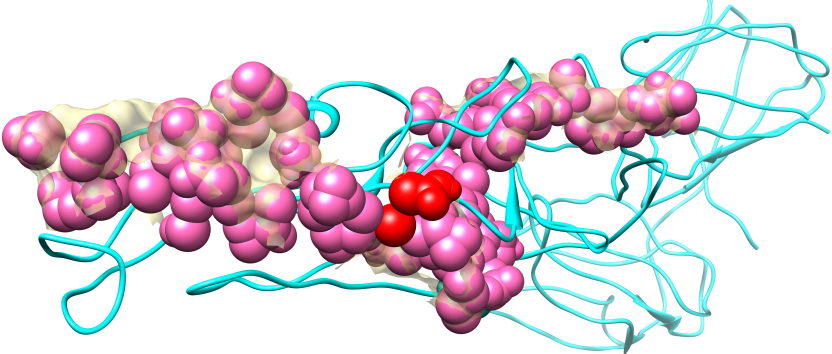


**S Figure 1:** Predicted B cell epitopes in dengue virus envelope protein are shown in hot pink, red indicates epitopic residue ASN 67 which is involved DC-SIGN binding.


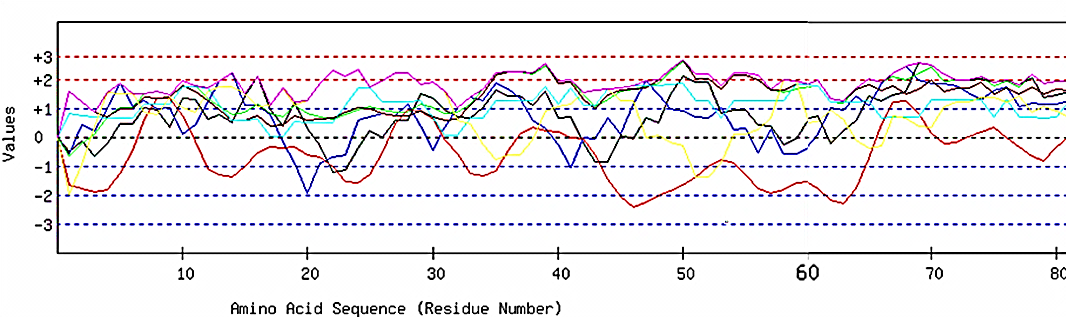


**S Figure 2**: Physiochemical properties of B cell epitope: The peak of the amino acid residue segment above the threshold value (2 to 2.5) is considered as predicted B cell epitope.

**Colors**: Hydrophilicity, turns, surface, Flexibility, polar, accessibility, antigenic.
