# Supplementary material for: Interaction and Inhibition of Dengue Envelope Glycoprotein with Mammalian Receptor DC-Sign, an In-Silico Approach
Source: PLoS One. 2013 Mar 18;8(3):e59211. doi: 10.1371/journal.pone.0059211 (PMC3601059; doi:10.1371/journal.pone.0059211)
Supplement: File S2 — Includes Tables S1 and S2. (DOCX) [file pone.0059211.s002.docx]

**S Table 1: Den-E-DC-SIGN complexes:** Buried surface area, solvation free energy (**Δ^i^G)** and hydrogen bonds in interfacing residues of Den-E-DC-SIGN complexes are listed. Models are selected by 3 step filtering process.

| Model No. | Buried area, Å^2^ | Δ^i^G, kcal/mol | NHB |
| --- | --- | --- | --- |
| 2 | 658.4 (37%) | -4.2 (203%) | 5 (71%) |
| 5 | 897.7 ( 38%) | -3.5 (213%) | 8 ( 67%) |
| 7 | 711.8 ( 37%) | -0.3 ( 6%) | 16(75%) |
| 8 | 554.1 ( 34%) | -3.2 (236%) | 4 ( 80%) |
| 9 | 620.0 ( 34%) | -4.1 (592%) | 5 (100%) |
| 10 | 934.8 ( 41%) | -3.5 (758%) | 8 (100% |
| 16 | 754.4 ( 39%) | -2.6 (301%) | 11 (100%) |
| 18 | 929.9 ( 38%) | -3.8 (265%) | 7 ( 88%) |
| 20 | 633.2 ( 34%) | -3.8 (380%) | 4 ( 67%) |
| 23 | 413.8 ( 30%) | -0.3 ( 5%) | 5 ( 50%) |
| 24 | 573.4 ( 34%) | -2.1 ( 57%) | 6 ( 86%) |
| 25 | 653.1 ( 35%) | -3.8 (343%) | 4 ( 80%) |
| 27 | 908.0 ( 39%) | -4.7 (1792%) | 9 ( 75% |

**S Table 2: SelectedDen-E-2G12 complexes:** Buried surface area, solvation free energy (**Δ^i^G)**,hydrogen bonds and salt bridges present in interfacing residues of Den-E-2G12 antibody complexes are listed. Models are selected by 3 step filtering process.

| Modl No. | Chain ID | Buried area, Å^2^ | Δ^i^G, kcal/mol | NHB |
| --- | --- | --- | --- | --- |
| 1 | A+H | 534.3 ( 9%) | -1.0 ( 6%) | 7 ( 30%) |
|  | A+L | 138.8 ( 2%) | -1.9 ( 11%) | 2 ( 9%) |
| 2 | A+H | 626.2 ( 9%) | 1.2 ( 7%) | 3 ( 13%) |
|  | A+L | 468.5 ( 7%) | -3.3 ( 21%) | 2 ( 9%) |
| 3 | A+H | 415.2 ( 7%) | 0.6 ( 3%) | 3 ( 15%) |
|  | A+L | 464.8 ( 8%) | -4.3 ( 22%) | 4 ( 20%) |
| 4 | A+H | 591.4 ( 9%) | 2.6 ( 18%) | 7 ( 30%) |
|  | A+L | 326.9 ( 5%) | -2.5 ( 17%) | 2 ( 9%) |
| 5 | A+H | 521.9 ( 9%) | 1.0 ( 6%) | 10 ( 37%) |
|  | A+L | 106.7 ( 2%) | -1.2 ( 8%) | 2 ( 7%) |
| 6 | A+H | 426.5 ( 7%) | 0.5 ( 4%) | 6 ( 27%) |
|  | A+L | 426.5 ( 7%) | -0.6 ( 5%) | 3 ( 14%) |
| 7 | A+H | 442.3 ( 8%) | -0.4 ( 2%) | 6 ( 27%) |
|  | A+L | 120.5 ( 2%) | -1.4 ( 8%) | 1 ( 5%) |
| 9 | A+H | 568.7 ( 10%) | -1.6 ( 9%) | 6 ( 29%) |
|  | A+L | 117.3 ( 2%) | -1.4 ( 7%) | 2 ( 10%) |
| 12 | A+H | 389.0 ( 7%) | -1.1 ( 7%) | 8 ( 35%) |
|  | A+L | 303.7 ( 5%) | -2.4 ( 14%) | 1 ( 4%) |

**S Table 3**: Hydrogen bonds and salt bridges present at the interface of Den-E-DC-SIGN complex

| Hydrogen bonds | | | |
| --- | --- | --- | --- |
| ## | **Den-E** | [**Dist. [Å]**](javascript:openWindow('pi_ipage_atmdist.html',400,250);) | [**DC-SIGN**](javascript:openWindow('pi_ipage_atom2.html',400,250);) |
| 1 | A:THR  68[ N  ] | 3.69 | B:ASN 311[ O  ] |
| 2 | A:LYS 247[ NZ ] | 2.87 | B:GLU 347[ OE1] |
| 3 | A:LYS 247[ NZ ] | 2.74 | B:ASN 349[ OD1] |
| 4 | A:HIS 244[ NE2] | 3.01 | B:GLU 354[ OE2] |
| 5 | A:THR  70[ N  ] | 3.16 | B:SER 360[ OG ] |
| 6 | A:LYS 247[ NZ ] | 2.76 | B:ASP 366[ OD2] |
| 7 | A:LEU  65[ O  ] | 2.71 | B:GLN 274[ NE2] |
| 8 | A:THR  68[ O  ] | 3.25 | B:PHE 313[ N  ] |
| 9 | A:THR  70[ O  ] | 3.37 | B:SER 360[ OG ] |
| 10 | A:ARG  73[ O  ] | 3.26 | B:ARG 345[ NH2] |
| 11 | A:CYS  74[ SG ] | 3.44 | B:ARG 345[ NH1] |
| 12 | A:CYS  74[ SG ] | 3.69 | B:ARG 345[ NH2] |
| 13 | A:GLN  77[ OE1] | 3.20 | B:ARG 345[ NH1] |
| 14 | A:GLY 104[ O  ] | 3.15 | B:ARG 345[ NH2] |
| 15 | A:CYS 105[ SG ] | 3.39 | B:ARG 345[ NH2] |
| 16 | A:NAG1395[ O7 ] | 3.26 | B:ASN 272[ N  ] |
| Salt Bridges | | | |
| 1 | A:LYS 247[ NZ ] | 2.87 | B:GLU 347[ OE1] |
| 2 | A:LYS 246[ NZ ] | 2.74 | B:GLU 347[ OE2] |
| 3 | A:LYS 247[ NZ ] | 3.98 | B:GLU 354[ OE1] |
| 4 | A:HIS 244[ NE2] | 3.01 | B:GLU 354[ OE2] |
| 5 | A:LYS 247[ NZ ] | 2.89 | B:ASP 366[ OD1] |
| 6 | A:LYS 247[ NZ ] | 2.76 | B:ASP 366[ OD2] |
| 7 | A:GLU  71[ OE2] | 3.58 | B:ARG 345[ NE ] |

**S**

**Table 4**: Hydrogen bonds and salt bridges present at the interface of Den-E-2G12 complex

| Hydrogen bonds | | | |
| --- | --- | --- | --- |
|  | **Den-E** | **Dist. [Å]** | **2G12** |
| 1 | A:THR  68[ H  ] | 2.31 | H:THR  56[ O  ] |
| 2 | A:THR  70[ H  ] | 1.84 | H:ARG  58[ O  ] |
| 3 | A:LYS 247[ HZ1] | 1.64 | H:ASP 106[ OD1] |
| 4 | A:LYS 247[ HZ3] | 1.72 | H:ASP 106[ OD2] |
| 5 | A:THR  68[ O  ] | 2.19 | H:ARG  58[ H  ] |
| 6 | A:ASN  67[ OD1] | 1.79 | H:ARG  58[HH11] |
| 7 | A:ASN  67[ OD1] | 1.92 | H:ARG  58[HH21] |
| 8 | A:THR  70[ O  ] | 1.77 | H:LYS  65[ HZ2] |
| 9 | A:GLU  71[ OE1] | 1.62 | H:LYS  65[ HZ1] |
| 10 | A:GLY 102[ H  ] | 1.71 | L:GLU  30[ OE2] |
| 11 | A:LYS 246[ HZ1] | 1.66 | L:TYR  94[ O  ] |
| 12 | A:LYS 246[ HZ2] | 1.91 | L:SER  95[ O  ] |
| 13 | A:LYS 246[ HZ3] | 1.73 | L:SER  95[ OG ] |
| 14 | A:GLY 104[ O  ] | 1.69 | L:VAL   2[ H2 ] |
| 15 | A:GLY 102[ H  ] | 1.71 | L:GLU  30[ OE2] |
| 16 | A:LYS 246[ HZ1] | 1.66 | L:TYR  94[ O  ] |
| 17 | A:NAG1395[ O7 ] | 2.14 | H:ARG  58[HH22] |
| Salt bridges | | | |
| 1 | A:HIS 244[ ND1] | 3.75 | H:ASP 106[ OD1] |
| 2 | A:LYS 247[ NZ ] | 2.56 | H:ASP 106[ OD1] |
| 3 | A:LYS 247[ NZ ] | 2.59 | H:ASP 106[ OD2] |
| 4 | A:GLU  71[ OE1] | 2.62 | H:LYS  65[ NZ ] |
| 5 | A:HIS 244[ ND1] | 3.75 | H:ASP 106[ OD1] |
